# Pertuzumab in Metastatic Breast Cancer

**DOI:** 10.6004/jadpro.2012.3.6.5

**Published:** 2012-11-01

**Authors:** Rachana Patel, Jill S. Bates

The human epidermal growth factor family of receptors (HER1/EGFR, HER2, HER3, and HER4) plays an important role in the proliferation, differentiation, and transformation of tumor cells (Gianni et al., 2010). The receptors are highly expressed in many solid tumor types, including breast cancer. Approximately 15% to 30% of breast cancers overproduce the growth-promoting protein HER2/*neu*. These tumors tend to grow fast and are generally more likely to recur than tumors that do not overproduce HER2.

The introduction of the humanized monoclonal antibody trastuzumab (Herceptin), which suppresses HER2-mediated signaling by binding to the extracellular domain of the HER2 protein, has dramatically improved outcomes for patients with HER2-positive breast cancer and changed the natural course of HER2-positive disease (Baselga et al., 2012). However, as most patients with HER2-positive metastatic breast cancer will eventually progress on trastuzumab regimens, additional interventions will be required to extend the life expectancy of these patients. Dual antibody therapy targeted toward HER2 represents an important treatment option.

## Pharmacology

Pertuzumab (Perjeta) is a recombinant, humanized monoclonal antibody that targets the extracellular dimerization domains of HER2 and thereby blocks ligand-dependent heterodimerization of HER2 with other HER family members, including EGFR, HER3, and HER4 (Gianni et al., 2010). As a result, pertuzumab inhibits ligand-initiated intracellular signaling through two major signaling pathways: mitogen-activated protein (MAP) and phosphoinositide 3-kinase (PI3K; Genentech, 2012). Inhibition of these signaling pathways can result in cell growth arrest and apoptosis (Genentech, 2012).

Preclinical models have shown that pertuzumab inhibits tumor growth in the absence of HER2 overexpression, unlike trastuzumab. Because pertuzumab and trastuzumab bind to different epitopes of HER2, it is hypothesized that the complementary mechanisms of action of the two agents could lead to synergistic antitumor effects when given in combination (Baselga et al., 2010).

Pertuzumab is indicated in combination with trastuzumab and docetaxel for the treatment of patients with HER2-positive metastatic breast cancer (Genentech, 2012). The initial dose of pertuzumab is 840 mg administered as a 60-minute IV infusion, followed every 3 weeks thereafter by 420 mg administered as a 30- to 60-minute infusion.

## Key Clinical Studies

Clinical trials of pertuzumab for the treatment of metastatic breast cancer have included administration of the agent alone or in combination with trastuzumab. A phase II trial of pertuzumab in patients with HER2-negative metastatic breast cancer was conducted to assess the antitumor activity and safety profile of pertuzumab monotherapy (Gianni et al., 2010). This was an international, multicenter, open-label randomized study in which 79 patients were randomly assigned to a loading dose of 840 mg on day 1 followed by 420 mg every 3 weeks (arm A) or no loading dose and 1,050 mg every 3 weeks (arm B). Treatment was continued until disease progression, unacceptable toxicity, or death.

Of the 78 patients evaluable for efficacy, 2 patients in arm A (n = 41) had partial responses, and 18 patients (44%) experienced stable disease lasting > 12 weeks. In arm B (n = 37), Stable disease was observed in 14 patients (38%). Overall, 6 of 78 patients responded or had stable disease > 6 months. Both dose levels of pertuzumab were generally well tolerated. The most frequent toxicities seen in both arms were grade 1 or 2 diarrhea, asthenia, nausea, and vomiting. Eight patients experienced drops in left ventricular ejection fraction (LVEF) of > 10%; however, all cases were asymptomatic. Pharmacokinetic data supported a fixed dose of pertuzumab every 3 weeks.

In another multicenter, open-label, single-arm phase II trial, the efficacy and safety profile of the combination of pertuzumab and trastuzumab were assessed in patients with HER2-positive breast cancer whose disease had progressed during prior trastuzumab-based therapy (Baselga et al., 2010). Patients received trastuzumab weekly (4 mg/kg loading dose, then 2 mg/kg every week) or every 3 weeks (8 mg/kg loading dose, then 6 mg/kg every 3 weeks) and pertuzumab every 3 weeks (840 mg/kg loading dose, then 420 mg every 3 weeks). Of 66 patients, 5 patients (7.6%) experienced a complete response, 11 patients (16.7%) experienced a partial response, and 17 patients (25.8%) experienced stable disease of > 6 months. Median progression-free survival was 5.5 months. The pertuzumab/trastuzumab combination was generally well tolerated, and the most frequent adverse events were mild to moderate.

In a study conducted by Cortés et al., 29 patients with HER2-positive breast cancer whose disease progressed during prior trastuzumab-based therapy received pertuzumab (840 mg loading dose, then 420 mg every 3 weeks) until progressive disease or unacceptable toxicity (Cortés et al., 2012). Seventeen patients with disease progression continued to receive pertuzumab (at the same dose) with the addition of trastuzumab (4-mg/kg loading dose and then 2 mg/kg weekly or 8-mg/kg loading dose and then 6 mg/kg every 3 weeks). All 29 patients enrolled for pertuzumab monotherapy experienced disease progression. The objective response rate (ORR) and clinical benefit rate (CBR) were 3.4% and 10.3%, respectively, during pertuzumab monotherapy. With the addition of trastuzumab, the ORR and CBR were 17.6% and 41.2%, respectively. Progression-free survival was 17.4 weeks with combination therapy vs. 7.1 weeks with pertuzumab monotherapy.

An international, randomized, double-blind, placebo-controlled phase III trial was conducted in approximately 800 patients with HER2-positive metastatic breast cancer. Patients received either placebo plus trastuzumab and docetaxel or pertuzumab plus trastuzumab and docetaxel (Baselga et al., 2012). The median progression-free survival was 12.4 months in the control group, as compared with 18.5 months in the pertuzumab group (hazard ratio for progression or death, 0.62; 95% confidence interval = 0.51 to 0.75; * p* < .001). The safety profile was generally similar in the two groups.

## Adverse Events

The most commonly reported adverse events that have occurred in patients on pertuzumab include diarrhea, alopecia, neutropenia, nausea, fatigue, rash, and peripheral neuropathy (Table 1). Most adverse events were grades 1/2. The most common adverse events of grade 3 or higher were neutropenia, febrile neutropenia, and leukopenia (Genentech, 2012).

**Table 1:**
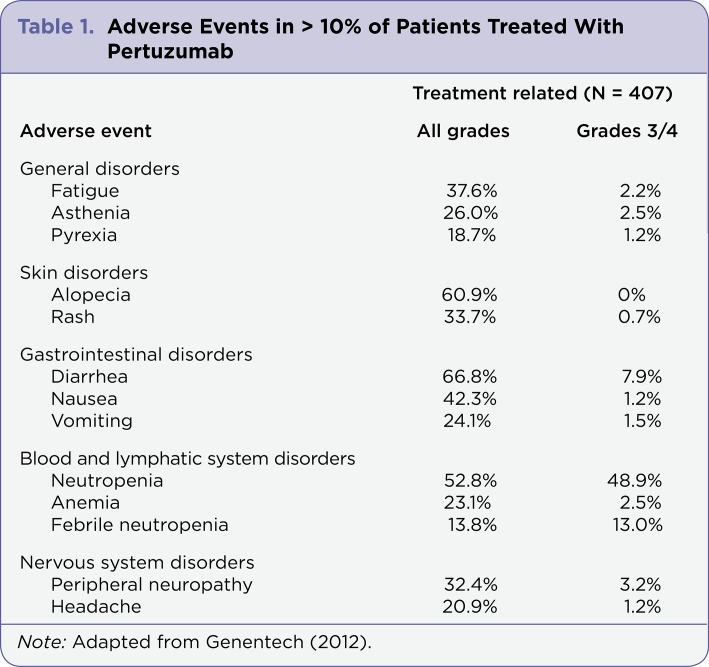
Table 1. Adverse Events in > 10% of Patients Treated With Pertuzumab

Other significant adverse events that have been reported with pertuzumab include left ventricular dysfunction, infusion-associated reactions, hypersensitivity reactions, and anaphylaxis. Pertuzumab is approved with a black box warning regarding embryo-fetal toxicity and birth defects. Patients should be advised of these risks as well as the need for effective contraception prior to starting pertuzumab.

## Role in Therapy

Treatment selection for metastatic breast cancer is guided by several factors, most importantly hormone receptor or HER2 expression as well as prior treatments. Cytotoxic chemotherapy is the initial treatment for metastatic breast cancer. Anthracyclines or taxanes are commonly used in first-line metastatic breast cancer (Smith, 2011). The humanized monoclonal antibody pertuzumab is the first in a new class of drugs, the HER dimerization inhibitors. Given that pertuzumab binds to a different epitope of the HER2 extracellular domain than trastuzumab, combination therapy with pertuzumab plus trastuzumab may result in more comprehensive blockade of HER2 signaling than can be achieved with trastuzumab alone (Baselga et al., 2010). Patients with HER2-overexpressing breast cancer who progress on trastuzumab may benefit from the addition of pertuzumab to trastuzumab therapy. Given its manageable adverse event profile, pertuzumab is a reasonable treatment option in patients with metastatic breast cancer.

## Implications for the Advanced Practitioner

Detection of HER2 protein overexpression is necessary for selection of patients appropriate for pertuzumab therapy because they are the only patients studied and for whom benefits have been shown. Patients being considered for treatment with pertuzumab should have LVEF checked at baseline and then every 3 months thereafter. Dosing should be held for at least 3 weeks if there is either a drop in LVEF to less than 40% or LVEF of 40%–45% with a 10% or greater absolute decrease below pretreatment values. If after a repeat assessment within approximately 3 weeks the LVEF has not improved, or has declined further, discontinuation of pertuzumab should be strongly considered (Genentech, 2012).

Dose adjustments are not needed in patients with mild (creatinine clearance 60–90 mL/min) or moderate (creatinine clearance 30–60 mL/min) renal impairment. No dose adjustment can be recommended for patients with severe renal impairment (creatinine clearance less than 30 mL/min) due to limited pharmacokinetic data. No clinical studies have been conducted to evaluate the effect of hepatic impairment on the pharmacokinetics of pertuzumab (Genentech, 2012).

## Summary

One third of patients with primary breast cancer will go on to develop metastatic breast cancer. The HER2-targeted monoclonal antibody trastuzumab has dramatically improved the prognosis for patients with HER2-positive metastatic breast cancer, but almost all patients will eventually progress on trastuzumab therapy. Pertuzumab is a humanized monoclonal antibody that inhibits tumor growth and survival by a novel mechanism of action-inhibition of dimerization of HER2 with other ligand-activated HER kinases. Combining anti-HER receptor monoclonal antibodies may result in an improved receptor blockage and a better clinical outcome.
